# Medical and Philosophical Intelligence

**Published:** 1815-03

**Authors:** 


### MEDICAL AND PHILOSOPHICAL INTELLIGENCE.

ROYAL SOCIETY.—On Thursday the 12th of January, the remainder of Mr. TRAVERS's Paper on the Way in which the Eye accommodates itself to the Sight of Objects at different Distances, was read. He considers the iris as muscular, and as connected with a kind of inner iris, or ring, which by its contraction increases the convexity of the lens. The eye, he conceives, is only acted upon by the stimulus of light. Hence, he believes, that the contraction of the iris is regulated entirely by the retina. The eye, in his opinion, is fitted naturally for viewing distant objects. When near objects are viewed, the iris contracts, in consequence of which the inner ring acts upon the lens, and increases its convexity. The sensation of fatigue he supposes owing to the over-action of the external muscles of the eye, and not to any fatigue in the iris itself.

---

On Tuesday, the 14th instant, was the Anniversary of the Hunterian Oration. Our readers are probably aware, that by the liberality of his two representatives, Dr. Baillie and Sir Everard Home, a fund is established for the celebration of this day, by an annual oration.

Sir Everard of course delivered the first oration, which was last year. Sir William Blizard was appointed orator for the present. He began with the usual topics in honour of the art, tracing it to the Trojan war, but commencing in England in a scientific form under Wiseman, the first, who, in this country, gave a systematic work on Surgery. He afterwards enumerated with equal candour, correctness, and propriety, the names of W. Sharpe, Hawkins, Bromfield, Pott, and a few others who have distinguished themselves as writers, or improvers in the operating part of the art; this gradually led him to introduce the name of Hunter, on which he dwelt with that eloquence which the subject seemed to inspire. His conclusion in reference to Mr. Cline, who is the next in turn to deliver the oration, was peculiarly happy. The audience was very numerous, and not less respectable or attentive, and the whole scene truly impressive.

---

The Royal Medical Society of Edinburgh having agreed to appoint a committee for the purpose of receiving the communications of members, and of others, through their medium, who may favour the society with interesting facts and experiments in medicine, or with unusual appearances in morbid dissection, beg leave earnestly to invite the members to transmit such communications to the society as soon as possible.

The committee, consisting of six extraordinary members resident in Edinburgh, together with the four presidents, ex-officio, will proceed immediately to consider such papers as may be transmitted to them, with an ultimate view to publication.

---

There is not a charitable institution in the metropolis, we might, perhaps, add in the kingdom, more deserving of notice, than the *City of London Truss Society.* Beginning with the slenderest means, and in the face of two others of long standing, without puffing or any other patronage than what has arisen from the manifestation of its usefulness, it has now risen to a degree of respectability, we had almost said wealth, which could not have been within the contemplation of its early supporters. An institution cannot, cannot, however, be wealthy which opens its arms to every application, requiring little more introduction than the distresses of the applicant. The following statement of the situation and occurrence of hernia, at different periods of life, has been extracted from the register of the patients relieved by the City of London Truss Society, within the short period of seven years:

In 7910 cases, 6523 were males, and 1387 were females.

| Males. | Females. | | | |
|---|---|---|---|---|
| 1469 | 14 | Left Inguinal | 4070 Inguinal | 4665 Single. |
| 2567 | 20 | Right Inguinal | | |
| 38 | 246 | Left Femoral | 595 Femoral | |
| 47 | 264 | Right Femoral | | |
| 2182 | 10 | Double Inguinal | | 2367 Double. |
| 36 | 139 | Double Femoral | | |
| 29 | 315 | Umbilical | | 388 |
| 10 | 34 | Ventral | | |
| | 1 | Obturator | | 1 |
| 17 | 26 | have undergone operations, all, except one, have been completely successful | | 43 |
| 63 | 72 | with Umbilical Hernia have been cured without Trusses | | 135 |
| 62 | 10 | with Prolapsus Ani | | 72 |
| | 236 | with Prolapsus Uteri | | 236 |
| 3 | | with Varix of the Abdominal Veins | | 3 |
| 6523 | 1387 | ——— 7910 | | 7910 |

| | | | | | | |
|---|---|---|---|---|---|---|
| 524 | patients were relieved with trusses under 10 years of age. | | | | | |
| 384 | ditto, | between | 10 | and | 20 | ditto. |
| 771 | ditto, | ——— | 20 | and | 30 | ditto. |
| 1286 | ditto, | ——— | 30 | and | 40 | ditto. |
| 1471 | ditto, | ——— | 40 | and | 50 | ditto. |
| 1420 | ditto, | ——— | 50 | and | 60 | ditto. |
| 988 | ditto, | ——— | 60 | and | 70 | ditto. |
| 347 | ditto, | ——— | 70 | and | 80 | ditto. |
| 38 | ditto, | ——— | 80 | and | 90 | ditto. |
| 2 | ditto, | ——— | 90 | and | 100 | ditto. |
| 7231 | | | | | | |

Six females each had umbilical and double femoral hernia; 1 female had large ventral and double femoral hernia; 1 female had umbilical and ventral hernia; 1 female had ventral and prolapsus uteri; 5 females had umbilical and single femoral hernia; 2 females had right inguinal congenital hernia; 2 females had left inguinal and right femoral hernia, 1 female had left femoral and right obturator hernia; 4 females had ventral and single femoral hernia; 1 female had double femoral and right inguinal hernia.

Eleven males each had left femoral and right inguinal hernia; 1 male had left inguinal and left femoral hernia; 7 males had left inguinal inguinal and right femoral hernia; 4 males had double inguinal and right femoral hernia; 3 males had double inguinal and left femoral hernia; 4 males had right inguinal and right femoral; 2 males had double inguinal and double femoral hernia; 2 males had ventral and right inguinal hernia; 1 male had ventral and left inguinal hernia; 1 male had umbilical and left inguinal hernia; 1 male had umbilical and left femoral hernia; 1 male had umbilical and right femoral hernia; 5 males had umbilical and double inguinal hernia; 1 male had double femoral and right inguinal hernia; 454 patients had congenital hernia; 1 male had very large varicose veins on the abdomen.

From the report of Mr. Taunton, Surgeon to the Society, it appears that this malady exists in one person in eight through the whole male population of this kingdom, and even in a much greater proportion among the labouring classes of the community, in manufacturing districts, particularly in those persons who are employed in weaving, or on the water as boatmen.

In one district in the western part of England, comprising 200,000 inhabitants, it has been ascertained, by actual observation, that upwards of one in five of the whole population labour under this malady.

---

Mons. Des Genettes has described, from his immediate observation, an effect of cold hitherto little known. It acts intensely on the brain and nerves, even when congelation has not affected any part of the body. Men who were marching with every appearance of the most decided and determined muscular power, suddenly complained of a veil covering their eyes, which soon assumed a wild aspect, and then became motionless. The whole muscular apparatus of the neck, and more especially the sterno-cleido mastoidei became rigid and fixed the head to the right or the left side; the rigidity gradually reached the trunk, the inferior extremities bent, and the unfortunate sufferers fell presenting all the symptoms of catalepsy or epilepsy. This dreadful spectacle was frequent on the retreat from Moscow.

---

The following formula is recommended by Mr. J. Wilson, as an excellent application for the cure of itch.

℞. Decoct. Veratri, ℥ viij.

Hydrarg. Oxymuriat. ʒj.

Potassæ. Nitrat. ʒ ij.

Ammoniæ. Muriatis, ℥ij. M. ft. Lotio frequenter utenda.

---

Messrs. Majendie and Breshet have recently succeeded in affecting a dog with rabies, by inoculating him with the saliva of a man labouring under hydrophobia.

---

A case of exudation of blood by the cutaneous pores has lately been stated to the Societé Medicale d'Emulation, by Dr. F. C. Caizergues. The hæmorrhage was active, and consequent on an attack of nephritic colic.

An

*An Historical Fragment on the Croup;* by J. V. E. Vaidy, Army Physician.—Many physicians have long thought, that the croup was unknown in Europe before the 18th century. They founded their opinion principally on the silence of writers anterior to that epoch; but some resemblance has been traced within these few years, between this cruel disease and that of which Baillon speaks, under the name of *affectio orthopnoica.* But no one has quoted an author farther back than Baillon. I partook of the general uncertainty in this respect, when I read, for the first time, the beautiful treatise of Galen *De Partibus Affectis.* I was struck with a passage which is at the beginning of this treatise, and of which I shall give the translation:—"In a little boy, who, in coughing had thrown up a thick and viscous tunic; we conjectured, that it was the interior substance of the larynx, when it forms the epiglottis. The child became well, though we did not expect it; but the voice was changed." Does not this passage afford all the characters of croup?—the subject is young—he throws up, in coughing, a portion of a membrane—the disease is of a very dangerous nature, as Galen had not much hope, and the voice was changed. That an inflammatory disease should shew itself of a sudden in a country where it was unknown before, always appeared to me a great exception to the fundamental laws of pathology. In short, all the diseases which have really appeared in Europe, at various periods, such as small pox, plica, syphilis, are cutaneous diseases; some of which are evidently contagious, and the others are supposed to be contagious. The croup, on the contrary, follows the course of common inflammatory diseases, it is sometimes epidemic, more often sporadic; and has never been known to be propagated by contagion. It follows from this comparison, that the croup bears no resemblance to the diseases, the first appearance of which can be brought to any determined period; and that it only offers the character of simple inflammation. This conclusion agrees by induction with an historical document of incontestible authority.

The other articles which we conceived most interesting, and promised for a future number, have, we find, been copied from our Journal. They are, Dr. Hossack on the stopping Hæmorrhage by Exposure to Air, and Mr. Weatherhead's Account of a Contagious Erysipelas; see our number for June last. We cannot help wishing, that the editors of the French Journals would be still more attentive to us. We refer to the continuance of their cruel experiments on living animals to ascertain the process of vomiting, and their ill-conducted enquiries concerning the itch insect. In our next number, we shall shew how much they might have learned concerning the black cataract from Mr. Stevenson's last edition.

---

*State of Medicine in China;* by M. Page, of Orleans.—The Chinese employ emetics and purgatives but very rarely; clysters are almost never used, because they regard them as too European, but but they make a free use of cordials. The importation of opium is prohibited under pain of death.

The Chinese, in the treatment of the itch and eruptive diseases, employ camphor and cinnabar also, with sulphur dissolved in woman's milk. They make use of borax in inflammations of the throat; it is reduced into powder, and blown upon the diseased part. They borrowed the use of the bark from the Jesuit missionaries.

They were acquainted with inoculation long before us. They practise it in general by introducing into one of the nostrils cotton imbibed with variolous matter: the cotton is allowed to remain twelve hours, and in seven days at latest the disease appears.

Like most Indian nations, they make a free use of aphrodisiacs, baths, and mineral waters. They have springs saturated with alum and iron, but the greater number contain sulphur. Their physicians are not able to analyse them. Chemistry, as well as natural history, is in its infancy at China. But the Chinese have the good fortune to possess a species of *mesmerism* or animal magnetism, as practised by certain sects of illuminati in Germany. The Chinese literati strive to put down this sect by ridicule; but they, nevertheless, find proselytes daily, to what they are pleased to call the *science of sciences.*

The Chinese are not acquainted with the making of bread, for which they substitute boiled rice or maize: their wine is a strong liquor extracted from honey or fermented rice. They do not drink either coffee or chocolate: they have delicious melons, the species of which is unknown to us, some very delicate kinds of small onions, and several delicious plants; but they have no olives, strawberries, gooseberries, or potatoes.

The diseases of stone and gravel are wholly unknown to the Chinese, in consequence, as they tell us, of the great quantity of tea which they drink.

---

*Account of some Electrical Phenomena recently communicated to the Royal Society of Edinburgh.*—In the year 1767, M. de Saussure, M. Pictet, and M. Jalabert ascended to Mount Breven, which is situated nearly in the middle of the valley of Chamouni, and almost exactly opposite to Mount Blanc. Their object was to take a general view of the form and position of the glaciers which descend from Mount Blanc. When they reached the summit, M. Jalabert began to take a drawing of the glaciers, M. Pictet was engaged in the geographical part, and M. de Saussure was preparing to make his experiments on natural and artificial electricity. When M. Pictet was laying down upon his plan some particular mountains by means of a graphometer, he had occasion to ask the names of them from the guides, and was therefore obliged to point out the individual mountains with his finger. Every time that he raised his hand for that purpose, he felt at the extremity of his finger a kind of tremulous and prickly sensation, similar to that which is experienced upon presenting the finger to a globe of glass highly electrified. M. Pictet soon perceived the origin of this phænomenon. He observed a stormy cloud in the middle region of Mount Blanc, directly opposite to Mount Breven, and it instantly occurred to him that he had been affected with the electricity of the cloud. He then requested MM. de Saussure and Jalabert to make the same experiment; and, as soon as they extended their hands, they experienced a similar sensation, which they described as resembling a number of small electric sparks; but fearing that they might be seduced by their imagination, they made their guides and their servants stretch out their hands, and they felt the same sensation. The electricity of the atmosphere having now begun to increase, the sensation became stronger, and was even accompanied with a kind of whistling noise. M. Jalabert, who had a gold band upon his hat, was alarmed with a buzzing noise about his head; and having taken off his hat, and put it successively upon the heads of Pictet and Saussure, they heard distinctly the same sound, and even obtained sparks from the golden button of the hat. The thunder cloud had now moved across the valley, and was directly above their heads. The thunder was loud, and the flashes of lightning so frequent, that the travellers found it prudent to descend about twenty or thirty yards, where no electricity could be perceived. The guides, however, were so much delighted with the experiments, that it was with great difficulty they were persuaded to abandon such a dangerous amusement. A shower of rain soon afterwards fell, and the storm ceased. The travellers re-ascended to the summit; and, although they elevated an electrical kite, they were unable to perceive any indications of electricity in the atmosphere.

Electrical phenomena, much similar to the above, were very recently observed by a party of Englishmen, when they were descending Mount Ætna during a storm of thunder and lightning. The following particulars were communicated by Mr. Gillies, surgeon, who not only read an account of the phenomena in a journal entitled *Specchio della scienza e Giornale Encyclopedica de Sicilia*, which was published in July 1814, but received an account of them from Mr. Tupper, one of the party, coinciding in every respect with that which was given in the Sicilian journal. On the 2d of June, Mr. Tupper and Mr. Lanfiar, accompanied by a guide, ascended Mount Ætna. During their descent, when at a little distance from the place called the English House, in the *regio deserta*, they were overtaken by a storm of thunder and lightning, accompanied with a heavy fall of snow; and while running over an extensive field of snow, they were struck by a flash of lightning, from which, however, they experienced no serious injury. Mr. Tupper felt a painful sensation in his back, which gradually ascended towards his head, and occasioned a sensation as if his hair was moving, an effect exactly similar to that which is produced either when a person is electrified upon an insulated support, or when his head is presented to a prime conductor of an electrifying machine.

This sensation induced Mr. Tupper to raise his hand to his head, upon which he was surprised to hear a buzzing noise proceeding from his finger. Upon raising and extending his arm, the noise still continued; but, upon moving his hands and fingers in different directions, and with different degrees of velocity, he found that he could produce, at pleasure, a great variety of harmonic sounds, differing in their tone as well as in their intensity, and so loud as that they could be distinctly heard at the distance of forty feet. The Sicilian guide witnessed these phenomena with extreme dismay; and imagining that Mr. Tupper produced the sounds in virtue of some supernatural power, he immediately began to cross himself, and invoked the protection of his saint. His alarm, however, speedily subsided, when, upon being desired to elevate his own arm, he found it as musical as that of Mr. Tupper. Mr. Lanfiar, who was a little behind the rest of the party, now joined them, and found that his fingers possessed a similar property. In the course of five minutes, reckoning, we presume, from the arrival of Mr. Lanfiar, their fingers lost their acoustic property, the cloud having by this time passed to a considerable distance. Mr. Tupper had received an injury in his left shoulder joint by a fall from his horse; but he never experienced any return of the pain after the copious electrization which he received on Mount Ætna.—*Phil. Mag.*

---

*Report of the Hospitals for Small Pox Inoculation and Vaccination*, at their annual meeting on the 22d instant, his Royal Highness the DUKE of KENT in the chair.

NATURAL SMALL POX.

| | | |
|---|---|---|
| Patients from the 26th Sept. 1746, to 1st Jan. 1814 | 22,470 | |
| During the year 1814 | 79 | |
| | | 22,549 |

VARIOLOUS INOCULATION.

| | | |
|---|---|---|
| Patients from the 26th Sept. 1746, to 5th May, 1808 | 47,363 | |
| In-patients from 5th May, 1808, to 1st Jan. 1814. | 481 | |
| ——— during the year 1814, (3 mothers of infants under 5 years of age, were admitted, on payment for their own board) | 27 | |
| | | 47,871 |

VACCINATION, begun 21st January, 1799.

| | | |
|---|---|---|
| From 21st Jan. 1799, to the 1st Jan. 1814. | 31,660 | |
| During year 1814, out-patients 1671; in-patients 8 | 1,679 | |
| | | 33,339 |
| Total | | 103,759 |

Large, however, as the benefits conferred by these hospitals have been from their first foundation, they were far from proving adequate to the wishes of the benevolent supporters. The short seclusion required even for passing through the process of inoculation, was inconvenient to the labouring poor; and children at the breast could only be inoculated as out-patients. The discovery of Vaccination opened a new and infinitely more desirable prospect of superseding superseding this terrible disease. Nor were the medical officers of the hospital backward in proving, by safe and well-conducted experiment, the real value of a blessing so congenial with the institution. The result of the whole was published as soon as it could be ascertained; and from these reports the new practice met with that early reception which it would otherwise have attained with greater difficulty.

From that time every encouragement has been given to vaccination. But it could not be expected, after the high commendation which, for more than fifty years had been bestowed on inoculation, that the impression in its favour could be immediately effaced, nor was it prudent to form a hasty decision on so important a concern. For a few years, therefore, the practice of inoculation was continued to children, too young to be received into the house, and whose parents could not reconcile their minds to the new practice. But after due experience, the governors thought it right to discontinue the inoculation of small-pox beyond the walls of the hospital, into which grown persons are admitted without expense, and children also, with their mothers, who pay a small compensation for their own board.

The following very interesting occurrences at the above hospital are worthy of being recorded:—Ann Smith, a married woman, aged 22 years, in the eighth month of her pregnancy, was admitted with confluent small-pox on the 5th November, 1814, on the 9th day of the fever, and 6th of the eruption. Notwithstanding the severity of the distemper, and the unfavourable situation of the patient in other respects, she passed through the whole with safety, and even without a premature birth. She continued in the hospital till the 16th of the following month, being about six weeks in all, when she was discharged perfectly recovered.

On the 14th January last, she was admitted into the City of London Lying-in Hospital, and safely delivered of a male child. On the 7th February, she attended at the Small-Pox Hospital, that her child might be vaccinated. The insertion shewing no marks of any effect produced, was repeated two days after; this second insertion appeared red for two days and then disappeared. On the 16th, therefore, 14 days from the first vaccination, the child was inoculated with small-pox matter. This also failed.

Here is an instance of a woman passing through a confluent casual small-pox at a more advanced period of pregnancy than has, we believe, been hitherto recorded without a premature birth, and even without the death of the child either before or a few days after delivery. It may also be fairly presumed, by the subsequent failure of vaccination and inoculation with recent matter, that the child passed through the disease *in utero*; though the mother, for more perfect security, has been advised to bring her child for a fourth insertion in a month or two from the last.

On the day on which the above Ann Smith was discharged, (December 16, 1814), Sarah Clement was admitted from the parish work-house of Christ's Church, Surry. She was a married woman, man, 22 years of age, and in the seventh month of her pregnancy: Her face was covered with a very bad confluent small-pox. It was the 9th day of the fever, and 7th of the eruption. On the 17th of January last, Mr. Wachsel, resident surgeon and apothecary to the institution, delivered her of a still-born male child with many pustules on the face and body, all in a state of maturity. On account of the thin state of the cuticle, the slough is exposed in each pustule. The subject is preserved in spirits by Mr. Wachsel. On the 11th of February, the mother was discharged perfectly recovered.

This child was born forty-one days after the woman had been seized with the symptoms of small-pox. But it is not easy to say at what period it died. If it really lived till a short time before birth, and there was nothing in its appearance to contradict such an opinion, the mother must have infected it twenty-one days after the commencement of the symptoms on herself, allowing about twenty days for the reception of the contagion by the child, to the period of beginning suppuration in the pustules.

We must not omit the history of two children, one of whom accompanied each of these women into the hospital. Ann Smith brought with her a son about nineteen months old, who had been with his mother during her whole illness. He was vaccinated immediately on his arrival, and passed through the whole process without any variolous symptoms. Sarah Clement brought with her a daughter, about eleven months old, who, two days before, (the 5th of the eruption on her mother) had been inoculated by Mr. Gilham. This child had also been constantly with its mother during her whole illness, and, like the former, went through the process of vaccination without any variolous symptoms.

The above histories contain several valuable facts, and the success of vaccination at so late a period, after constant exposure to small-pox in its worst form, is not the least interesting part.

---

Dr. Spurzheim has examined the skulls at the Museum in St. Bartholomew's Hospital, and also the heads of the boys in Christ's Hospital. At both he shewed sufficient discernment to justify the opinion, that craneology is really a science, and not inconsiderably advanced, considering how recently it has been discovered.

---

In the press, and shortly will be published, a translation of Orfila's interesting work on Animal, Vegetable, and Mineral Poisons.

---

In a few weeks will be published, a translation of Professor Laing's work on Military Surgery.

---

Mr. Accum has in the press, a Treatise on Gas Light, exhibiting a summary description of the apparatus and machinery best calculated for illuminating streets, houses, and public edifices, with carburetted hydrogen or coal gas; together with remarks on the utility, safety, and general nature, of this new branch of civil economy. The treatise will be illustrated with geometrical and perspective perspective designs, exhibiting the structure of the larger gas light apparatus, now successfully employed for lighting the streets and houses of this metropolis, as well as the smaller apparatus used for lighting manufactories and private establishments.

---

Mr. T. J. PETTIGREW, F.L.S. will commence his Spring Course of Lectures on Anatomy and Physiology on Friday the 10th of March, at half-past eight o'clock in the evening precisely, at his house, No. 3, Bolt-court, Fleet-street.

---

Dr. CLARKE and Mr. CLARKE will commence their next Course of Lectures on Midwifery, and the Diseases of Women and Children, on Monday, March 20th. The Lectures are read at Mr. Clarke's house, 21, Saville-row, Burlington Gardens, every day, from a quarter-past ten to a quarter-past eleven, for the convenience of students attending the Hospitals.

---

Dr. SQUIRE will, on Thursday the 16th of March, begin a Course of Lectures on the Theory and Practice of Midwifery, and the Diseases of Women and Children.

---

*To the Editor of the Medical and Physical Journal.*

SIR,

IN your Journal for last month, to my great surprize, I observed an imputation of *misrepresentation* against me from Mr. Woodham.

I have to declare, that I was perfectly uninfluenced by any intention of this kind, in my quotations or remarks respecting Mr. Woodham; and, moreover, upon an attentive review of them, it does not appear to me that any such construction can, with justice, be applied to them.

I have ever considered that candour, in disputed points, demands, that all quotations given for the purpose of remarking on, should be delivered *verbatim, and unmutilated*, the reader then being enabled to judge correctly of the merits on each side.

I have, in this instance, as I ever have done, and ever mean to do, adhered to this rule; Mr. Woodham, however, for what reason is best known to himself, has deviated from this rule, by substituting, in a note of mine, given by him, at page 91, a dash —— for a portion of a sentence, which I shall take the liberty of supplying: for "——," therefore, read "because it is the most familiar instance of fermentation."

The obvious answer to Mr. Woodham's *tender apprehension* respecting the evil consequences which might ensue in practice, by the adoption of my opinion of a prior existence of *variolous essence* in the human habit, and its expulsion, is the well-known fact, that, however the small-pox, either in the natural way, or by inoculation, is ameliorated or moderated by art, a recurrence of the disease is so exceedingly rare, as never to be expected or apprehended; and, consequently, admitting the truth of my opinion, that, even under these circumstances, this variolous essence itself is, ordinarily, ordinarily, entirely thrown off, or the habit so entirely purified of it, as to resist any future attacks of the disease.

With respect to the other remarks contained in the same paper, they appear to me totally uninteresting, and answering no useful purpose. I shall, therefore, decline noticing them, and observe, that, on my part, this controversy is finally closed. I am, Sir,

Your obedient humble Servant,

*Oxford, Feb.* 7, 1815. RICHARD WALKER.*

---

### REPORT OF DISEASES.

PULMONIC disease, always at this season prominent in town practice, is considerably abated. A case of variola in the confluent form, in a coal-heaver, aged thirty, terminated, as in such a subject might certainly be expected, fatally. The peculiarity in the case was, that the man, at different periods of his life, had been exposed to small-pox contagion, and particularly when a boy had been purposely sent amongst children labouring under the disease, without receiving the infection; and now, in mature age, became affected with the complaint, without being able to trace any exposure to the contagion. Three cases of continued fever, with severe pain in the head, but without delirium, occurred. They recovered under the use of purgatives, with saline antimonial medicines. In a case of obstinate pain in the head, in a boy aged eleven years, cupping produced speedy relief, when other remedies had failed.† In this instance, it is probable the passion of fear had some effect, for it operated to such a degree, as evidently to influence the capillary vessels, and impede the abstraction of blood; at least the cupper assigned it as a reason for being able to get only half the quantity directed to be taken away.

The case of chorea mentioned in the last Report is recovering under the tonic treatment. The editor has received a letter on this subject signed H. B. as follows: "Observing, in your Report of Diseases for January, that active purgatives had been tried in a case of chorea for two months without success, I beg leave to mention, that by their means I have succeeded in curing a girl, aged twelve, who had been affected with chorea to a considerable degree for upwards of three months. I observed, that, as the belly became less hard, and the stools less black and offensive, the convulsions diminished; and that when the stools became natural, they ceased altogether." We are obliged to the writer for these remarks, and assure him that our own experience bears ample testimony to the efficacy of the treatment he pursued. But the case reported last month resisted the plan; and chorea, like other diseases, in different subjects, sometimes requires a diversity of treatment.

---

* We have inserted this letter from a sense of justice to Mr. Walker: as the question at issue, has, we conceive, been amply discussed; we hope that the controversy will not be renewed.—ED.

† He is since completely recovered, and is now a fine healthy boy, although before, he had a sickly look, and was much debilitated.

METEOROLOGICAL

### METEOROLOGICAL REGISTER.

### *From January the 25th, to February the 23d,* 1815.

**Kept by C. BLUNT, Philosophical Instrument Maker, No. 38, Tavistock-Street, Covent-Garden.**

| Moon. | Day. | Wind. | Barometrical Pressure. Max. | Min. | Mean. | Temperature. Max. | Min. | Mean. | |
|---|---|---|---|---|---|---|---|---|---|
| | 26 | NE | 29·80 | 29·52 | 29 73 | 31 | 20 | 24 66 | Clear |
| | 27 | SW | 29· | 28 96 | 28·98 | 33 | 21 | 28 | Rain |
| | 28 | SW | 29·04 | 29·02 | 29·033 | 44 | 33 | 37 | Rain |
| | 29 | SW | 29·2 | 29·2 | 29·2 | 40 | 34 | 36 | Fair |
| | 30 | SW | 29·34 | 29·30 | 29 317 | 41 | 35 | 37 | Fair |
| | 31 | SW | 29·42 | 29·34 | 29·386 | 42 | 36 | 37·66 | Cloudy. Rain. |
| ◑ | 1 | SW | 29·45 | 29·40 | 29·42 | 45 | 37 | 39·66 | Rain |
| | 2 | N | 29·60 | 29 52 | 29·56 | 43 | 35 | 38·33 | Cloudy |
| | 3 | S | 29·60 | 29·56 | 29·536 | 49 | 38 | 42 | Rain |
| | 4 | S | 29 61 | 29·48 | 29·532 | 57 | 37 | 42·33 | Rain |
| | 5 | SW | 29·61 | 29·61 | 29 61 | 52 | 38 | 43·33 | Fair |
| | 6 | W | 29·63 | 29·60 | 29·616 | 48 | 36 | 41·33 | Rain |
| | 7 | W | 29·72 | 29·59 | 29·652 | 50 | 38 | 42·33 | Rain |
| | 8 | W | 29·70 | 29·55 | 29·612 | 50 | 38 | 43·33 | Cloudy |
| ● | 9 | SE | 29·97 | 29·66 | 29·746 | 45 | 36 | 41 | Cloudy |
| | 10 | E | 29·63 | 29·57 | 29 607 | 48 | 40 | 43 33 | Rain |
| | 11 | W | 29·50 | 29·37 | 29·432 | 50 | 38 | 45·66 | Rain |
| | 12 | W | 29·40 | 29·40 | 29·40 | 50 | 40 | 44 | Rain |
| | 13 | W | 29·60 | 29·50 | 29 533 | 50 | 36 | 43 66 | Fair |
| | 14 | W | 29·52 | 29 48 | 29 49 | 50 | 38 | 43·33 | Fair |
| | 15 | NW | 29·69 | 29 68 | 29·685 | 52 | 42 | 46 | Fair |
| | 16 | W | 29·56 | 29·54 | 29·545 | 52 | 36 | 46 | Rain |
| ◐ | 17 | NW | 29 9 | 29·57 | 29·757 | 52 | 35 | 44 | Fair |
| | 18 | SW | 30·26 | 29·68 | 29·883 | 50 | 39 | 45 | Rain Wind |
| | 19 | W | 29·30 | 29·30 | 29·30 | 51 | 44 | 46·33 | Cloudy |
| | 20 | W | 29·78 | 29·64 | 29·705 | 55 | 43 | 29·66 | Rain |
| | 21 | SW | 30·04 | 29·83 | 29·957 | 55 | 47 | 51·66 | Fair |
| | 22 | W | 30·16 | 30·16 | 30·156 | 55 | 47 | 51·66 | Cloudy. Fair. |

RESULTS.

Mean barometrical pressure of the month 29·5482
Maximum 30·26 wind at SW
Minimum 28·96 ——— SW

Mean temperature of the month 41·9487 deg.

Maximum 57, wind at S

Minimum 20, ——— NE

*Scale exhibiting the prevailing Winds during the Month.*

| N | NE | E | SE | S | SW | W | NW |
|---|---|---|---|---|---|---|---|
| 1 | 1 | 1 | 1 | 2 | 9 | 11 | 2 |

| | Mean barometrical pressure. | Mean temperature. |
|---|---|---|
| From the full moon on the 25th Jan. to the last quarter on the 1st Feb. | 29·274 | 33·386 |
| ——— last quarter on the 1st, to the new moon on the 9th | 29·573 | 41·46 |
| ——— new moon on the 9th, to the first quarter on the 17th | 29·554 | 45·372 |
| ——— first quarter on the 17th, to the full moon on the 23d | 29·793 | 47·577 |

MONTHLY

